# Ferroptosis in Cancer: Epigenetic Control and Therapeutic Opportunities

**DOI:** 10.3390/biom14111443

**Published:** 2024-11-13

**Authors:** Roberta Veglia Tranchese, Sabrina Battista, Laura Cerchia, Monica Fedele

**Affiliations:** Institute of Experimental Endocrinology and Oncology “G. Salvatore” (IEOS), National Research Council—CNR, 80131 Naples, Italy; robertaveglia91@hotmail.it (R.V.T.); sabattis@unina.it (S.B.); cerchia@unina.it (L.C.)

**Keywords:** ferroptosis, cancer therapy, epigenetic regulation, lipid peroxidation, therapeutic targets

## Abstract

Ferroptosis, an iron-dependent form of regulated cell death driven by lipid peroxidation, has emerged as a critical pathway in cancer biology. This review delves into the epigenetic mechanisms that modulate ferroptosis in cancer cells, focusing on how DNA methylation, histone modifications, and non-coding RNAs influence the expression and function of essential genes involved in this process. By unraveling the complex interplay between these epigenetic mechanisms and ferroptosis, the article sheds light on novel gene targets and functional insights that could pave the way for innovative cancer treatments to enhance therapeutic efficacy and overcome resistance in cancer therapy.

## 1. Introduction

Programmed cell death (PCD) is a fundamental process in embryogenesis, cell homeostasis, and immune response. It is essential for normal development, maintaining cellular balance, and preventing diseases, particularly cancer [1]. Several types of PCD have been identified, including apoptosis, pyroptosis, necroptosis, parthanatos, ferroptosis, and others [2]. Historically, the Stockwell group conducted a screen for small-molecule drugs to identify lethal compounds that selectively target cells expressing the oncogenic mutant *HRAS*. The most selectively lethal compound identified was a novel molecule named “erastin”. Interestingly, erastin did not exhibit the expected characteristics of apoptosis, as its lethality could be significantly suppressed by iron chelators and lipophilic antioxidants. This observation led the group to consider the possibility that erastin induced a regulated, but non-apoptotic, form of cell death [3]. Subsequently, a similar form of non-apoptotic, iron-dependent cell death was induced by the small-molecule RAS synthetic lethal 3 (RSL3) [4]. Then, Dixon and Stockwell showed that erastin acted by inhibiting the cystine/glutamate antiporter, namely system Xc^−^, leading to glutathione (GSH) depletion [5]. The term “ferroptosis” was coined in 2012 [6] to describe this novel iron-dependent, non-apoptotic form of PCD induced by erastin and RSL3.

**Figure 1:**
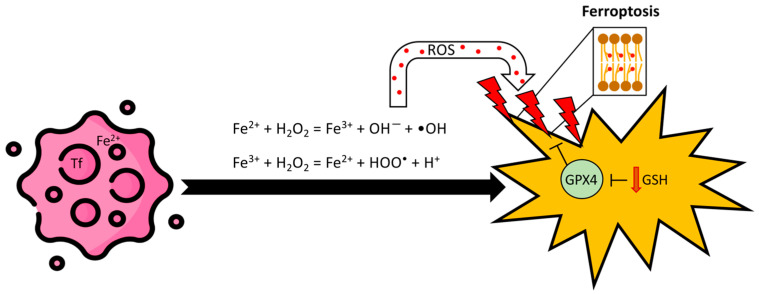
Schematic representation of the ferroptosis pathway in a cancer cell. Through Fenton reactions, the accumulation of free Fe^2+^ leads to the production of ROS, which oxidize membrane phospholipids, causing membrane rupture and cell death. The antioxidant enzyme GPX4 counteracts lipid peroxidation by utilizing GSH. Low levels of GSH impair GPX activity, thereby promoting ferroptosis. Tf, transferrin; Fe^2+^, ferrous ion; ROS, reactive oxygen species; GPX4, glutathione peroxidase 4; GSH, glutathione.

The ferroptotic cell exhibits unique morphological and biochemical characteristics. Typical is the abnormal mitochondrial morphology with small size, high membrane density, and reduced cristae surface, as well as rupture of the outer membrane. Lipids, with the aim to preserve the membrane integrity in the phospholipid bilayers, are susceptible to reactive oxygen species (ROS) attack. These species, mainly produced by iron overload, react with Polyunsaturated Fatty Acid (PUFA) and start a chain reaction to finally get lipid peroxides. On the other hand, the glutathione peroxidase (GPX) enzymes, mostly GPX4, using GSH as a co-substrate, reduce the burden of lipid peroxides to the corresponding alcohols. The reduction of GSH S-transferase (GST) levels and/or GPX4 activity results in lipid peroxide accumulation and eventually cell death for ferroptosis (Figure 1) [7].

Recent studies have highlighted the potential of inducing ferroptosis as an innovative cancer therapy, given that chemoresistant mesenchymal and dedifferentiated cancer cells are particularly susceptible to ferroptosis inducers [8,9,10]. Triggering non-apoptotic cell death can make cancer cells more sensitive to classical chemotherapy, overcoming chemoresistance. Moreover, ferroptosis may enhance tumor suppression, as multiple tumor suppressors have been shown to promote susceptibility to this form of cell death. Therefore, the pharmacological modulation of ferroptosis is crucial for cancer treatment, and ferroptosis may represent another important road to cancer cure [11]. Small-molecule drugs can initiate ferroptosis mainly in four ways: (i) by lowering intracellular GSH levels; (ii) targeting and inactivating GPX4; (iii) consuming GPX4 and endogenous antioxidant Coenzyme Q_10_ (CoQ_10_) via the SQS-mevalonate pathway; and (iv) inducing lipid peroxidation through an increased labile iron pool (LIP) or iron oxidation [12].

Currently, ferroptosis is widely acknowledged as a *bona fide* target for advancing treatment and prevention strategies for many diseases associated with ferroptosis, including cancer. However, the effectiveness of inducing ferroptosis depends on the specific genetic background of the cancer cells [13], and tumor cells can escape ferroptosis by triggering metabolic reprogramming [14]. Recently, iron-based nanocomposites have been developed, either alone or functionalized with chemotherapeutic drugs, to release both iron and chemotherapy agents into the tumor microenvironment. This strategy aims to elevate ROS levels, thereby enhancing ferroptosis [15]. Significant efforts have been dedicated to developing pharmacological agonists and antagonists to address these ferroptosis-related conditions [16]. Among them, small-molecule compounds are estimated to target only about 15% of proteins in the human proteome [17], and poor pharmacokinetics remains a major limitation in the further development of ferroptosis antagonists [16].

Epigenetic alterations in cancer are critical for the acquisition of a resistant phenotype and can control the expression of ferroptosis regulators or related pathways, leading to changes in cell sensitivity to ferroptosis inducers or cancer progression [18].

This article aims to examine the molecular mechanisms of ferroptosis, its epigenetic control, and the implications for developing novel cancer therapies, while also addressing the challenges and future directions in this field. To achieve this, an in-depth literature search in PubMed and Web of Knowledge was conducted, mainly focusing on publications from the last four years. The search combined keywords such as *programmed cell death*, *ferroptosis*, *cancer*, *epigenetic mechanisms*, and *ferroptosis-targeting drugs* to identify relevant studies.

## 2. Signaling Pathways Regulating Ferroptosis in Cancer

**Figure 2:**
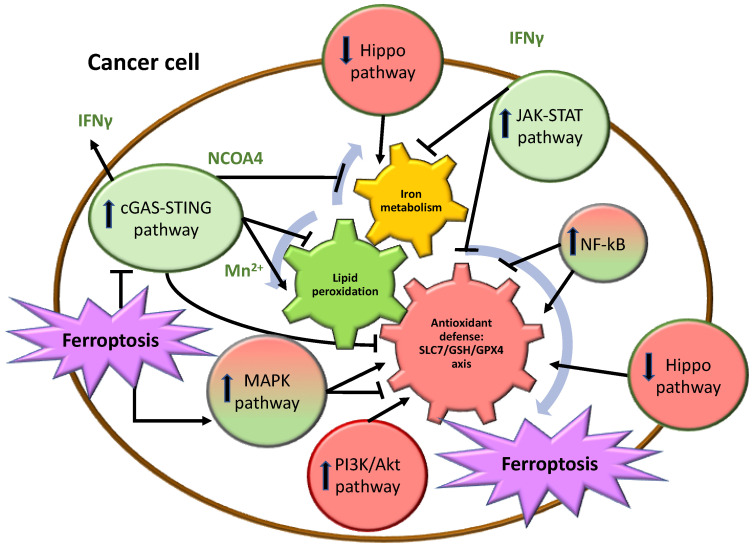
Schematic representation of the complex relationship among signaling pathways in a cancer cell. The three core pathways involved in the activation of ferroptosis are depicted in the middle as interconnected gears, each positively (↑) or negatively (⊥) regulated by a series of intracellular pathways that are either activated or inactivated in the tumor cell. These pathways are represented in red if they lead to inhibition of ferroptosis, green if they lead to activation of ferroptosis, and mixed red and green if they both activate and inhibit ferroptosis.

A complex interplay of pathways involving antioxidant defenses, lipid peroxidation, and iron metabolism, regulated by specific cancer cell signaling axes, orchestrates ferroptosis occurrence in cancer (Figure 2).

### 2.1. SLC7A11/GSH/GPX4 Axis

The light chain subunit solute carrier family 7 member 11 (SLC7A11; also known as xCT)-GPX4 signaling pathway is key in ferroptosis regulation, with GPX4 being a central player in this PCD form [19,20,21]. SLC7A11, part of the system Xc^−^, exchanges intracellular glutamate for extracellular cystine, critical for redox balance [22,23]. The system Xc^−^ consists of SLC3A2, a chaperon-stabilizing SLC7A11, which mediates cystine/glutamate transport. Cystine, reduced to cysteine, is a precursor for GSH, essential for detoxifying ROS with GPX4. This enzyme reduces peroxides to shield cells from ROS-induced damage using GSH, which is regenerated by GSH reductase [24,25]. Cancer cells upregulate SLC7A11 to manage elevated ROS levels. When GSH-dependent repair fails, lipid ROS accumulation induces ferroptosis (Figure 1) [24,26,27]. GPX4 inhibitors (e.g., RSL3, FIN56) [28] and Xc system inhibitors (e.g., erastin, sulfasalazine) trigger ferroptosis [29]. Lipid peroxide scavengers, such as ferrostatin-1 and Vitamin E, neutralize lipid peroxides [30]. IFNγ enhances ferroptosis under erastin or RSL3 treatment by promoting STAT1 binding to the *SLC7A11* promoter, depleting GSH, and increasing lipid peroxidation in tumor cells [10,31].

### 2.2. Iron Metabolism Pathway

Iron, a vital micronutrient, supports cell metabolism, growth, and proliferation [32] and is finely regulated through absorption, transport, uptake, and storage [33]. Dietary iron, present as Fe^3+^ in heme (red meat) or non-heme (cereals, vegetables), is reduced to Fe^2+^ by duodenal cytochrome b. Some iron is stored in ferritin, an iron reservoir, while other iron forms the cytoplasmic LIP or passes through ferroportin into the plasma, where it oxidizes to Fe^3+^ and binds to transferrin (Tf) for transport in the bloodstream [34].

Cancer cells, exhibiting “iron addiction”, rely heavily on iron for growth and are sensitive to iron depletion [35]. Excess iron can drive tumor development and lead to cell death via lipid peroxidation of the cell membranes, triggering ferroptosis [34]. Fe^2+^ promotes lipid peroxidation by generating ROS via the Fenton reaction (Figure 1) and acting as a lipid peroxidation cofactor [25]. Ferritin, particularly its heavy chain (FTH1), prevents ferroptosis by reducing ROS [36]. The Tf receptor TfR1, which imports iron [4], also serves as a ferroptosis marker due to its plasma membrane recruitment during the process [37].

### 2.3. Lipid Metabolism Pathway

Lipid metabolism significantly influences cell susceptibility to ferroptosis, particularly in cancer cells. Long-chain acyl-CoA synthases (ACSLs) are key in the biosynthesis and remodeling of PUFA-phosphatidylethanolamine (PE) in cellular membranes. They activate long-chain fatty acids (FAs) into fatty acyl-CoA esters, serving as substrates for synthesizing membrane phospholipids and triacylglycerols, or undergoing beta-fatty acid oxidation in mitochondria for energy. By mediating FA metabolism, ACSLs play a critical role in regulating endoplasmic reticulum (ER) stress, ferroptosis, drug resistance, and tumor inflammatory microenvironment, highlighting their significance in cancer progression [38].

Redox signaling in ferroptosis involves the engagement of PE with arachidonic acid (AA) or adrenergic acid (AdA) oxygenation. ACSL4 converts long-chain PUFAs to arachidonic CoA and adrenal CoA, increasing their oxidation susceptibility and leading to lipid peroxide formation, which can be incorporated into membrane phospholipids [25,38]. This process is necessary for producing ferroptosis death signals in cancer, representing a regulatory point for ferroptosis that future studies may further elucidate [39].

GPX4 reduces reactive lipid hydroperoxides to non-reactive alcohols using GSH, preventing toxic lipid radical formation through Fenton reactions with free iron (Figure 1). It uniquely converts PUFA hydroperoxides (PUFA-OOH) to alcohols (PUFA-OH), interrupting lipid peroxidation [40,41]. Insufficient GPX4-mediated lipid peroxide detoxification leads to lipid peroxide accumulation, resulting in degradation of the plasma and organelle membranes, which can drive tumor cell death (Figure 1).

### 2.4. JAK-STAT Pathway

The JAK-STAT pathway is a classical intracellular signal transduction pathway involved in various physiological and pathological processes, including cancer. Cytokines like interleukins and interferons, or hormones like leptin, bind to transmembrane receptors, triggering receptor oligomerization that allows JAK kinases to couple to and phosphorylate these receptors. This process promotes JAK activation and phosphorylation and dimerization of STAT proteins, which then translocate to the nucleus to regulate cytokine-responsive gene expression [41].

Interferon-gamma (IFNγ), produced by natural killer (NK) cells and T cells during tumor rejection, plays a critical role in tumor immunity by directly inducing apoptosis or autophagy in cancer cells. This increases their susceptibility to various death signals. Kong et al. found that IFNγ targets the system Xc^−^ in ferroptosis induced by erastin or RSL3 in hepatocellular cancer (HCC) cells. Reduced system Xc^−^ leads to GSH depletion and increased ROS production, making tumor cells more sensitive to lipid peroxidation and resulting in cell death [42]. Additionally, in ARPE-19 cells, IFNγ upregulates intracellular Fe^2+^ by inhibiting Ferroportin-1 and induces GSH depletion by blocking system Xc^−^. IFNγ also decreases GPX4 levels, heightening ferroptosis sensitivity via the JAK1-2/STAT1/SLC7A11 signaling pathway [43].

Propofol, a widely used anesthetic, induces ferroptosis and suppresses malignant behaviors in gastric cancer cells by modulating the miR-125b-5p/STAT3 axis [44]. As secondary messengers in the JAK-STAT pathway, STAT proteins regulate the expression of ferroptosis-related molecules, influencing the ferroptosis process. Recent studies suggest that the JAK-STAT pathway significantly impacts lipid peroxidation and ferroptosis by modulating redox systems and iron metabolism [45]. Given the complexity of this pathway, different stimuli can yield varying effects on ferroptosis, despite activating the same signaling pathway. Consequently, several agents targeting the JAK-STAT pathway show promising therapeutic potential for treating ferroptosis-related diseases, including cancer, in both cell and animal models.

### 2.5. PI3K/Akt Pathway

The interaction of specific ligands with receptor tyrosine kinases (RTKs) activates Phosphoinositide 3-kinases (PI3K), which phosphorylates phosphatidylinositol 4,5-bisphosphate (PIP2) to generate phosphatidylinositol 3,4,5-trisphosphate (PIP3) and activates protein kinase B (Akt). This pathway regulates various cellular functions, including apoptosis, growth, and glucose metabolism, and is often amplified in human malignancies [41,46,47].

The PI3K/Akt pathway is directly linked to ferroptosis as its activation influences multiple components involved in this PCD. For instance, Liu et al. found that in *IDH*-mutated gliomas, Akt activation triggers nuclear factor erythroid 2–related factor 2 (Nrf2)-mediated gene transcription of antioxidant proteins, leading to ferroptosis resistance. In glioma cells, increased Nrf2-driven transcription of antioxidant response elements (AREs) in the promoter regions of Nrf2 target genes, together with downregulation of pathways related to ferroptosis, counteracts ferroptosis. Therefore, targeting the Akt/Nrf2 pathway could enhance ferroptosis susceptibility in these tumor cells [48].

Mutations activating the PI3K/Akt/mTOR pathway are also associated with ferroptosis resistance. For example, mutations in the PI3KCA gene confer resistance in breast cancer cells [49].

### 2.6. cGAS-STING Pathway

In mammals, the cyclic GMP-AMP synthase (cGAS)—Stimulator of Interferon Genes (STING) signaling pathway mediates the immune response to DNA. This innate immune mechanism detects double-stranded DNA (dsDNA) from pathogens. Upon binding to dsDNA, cGAS converts ATP and GTP into cyclic GMP–AMP, a secondary messenger that activates STING on the ER membrane. This activation triggers a signaling cascade, leading to the production of immune mediators, including type I and type III interferons [50,51,52]. STING promotes ferroptosis through the regulation of type I interferon responses. It fosters erastin-induced ferroptosis by enhancing mitochondrial fusion via mitofusins (MFN1 and MFN2), which are crucial for mitochondrial dynamics. Genetic inactivation of the STING1-dependent mitochondrial fusion pathway limits the anticancer effects of ferroptosis activators in pancreatic cancer cells. Additionally, STING and mitochondrial ROS (mtROS) regulate each other in a positive feedback loop, where increased mtROS promotes mitochondrial STING accumulation, amplifying mtROS production and ferroptosis [53].

Nuclear receptor coactivator 4 (NCOA4) facilitates ferritinophagy, aiding in iron homeostasis. Aberrant NCOA4 activation can induce ferroptosis [54,55]. Activated STING interacts with NCOA4 in macrophages, provoking STING-mediated ferritin degradation, thereby supporting lipid peroxidation and ferroptosis [56]. Manganese (Mn^2+^) enhances cGAS-STING signaling, downregulating dihydroorotate dehydrogenase (DHODH), an enzyme that regulates mtROS production and lipid peroxidation, promoting ferroptosis in murine tumor cells [57].

Ferroptosis can also affect the cGAS-STING pathway. During herpes simplex virus-1 (HSV-1) infection, lipid peroxidation induces STING carbonylation, an irreversible modification that impairs protein function. GPX4 deficiency enhances lipid peroxidation, leading to STING carbonylation and disrupting its trafficking from the ER to the Golgi, suppressing STING activation [58].

### 2.7. The Hippo Pathway

The Hippo pathway is vital for regulating tissue development and organ size in mammals. It primarily consists of tumor-suppressing kinases, including microtubule-associated serine/threonine-protein kinase 1/2 (MST1/2) and large tumor suppressor kinase 1/2 (LATS1/2), alongside the oncogenic effectors YAP and TAZ. These components influence various cellular functions such as survival, migration, self-renewal, and differentiation [59]. Activation of the Hippo pathway leads to MST1/2 activation, which phosphorylates LATS1/2, resulting in the inactivation of YAP and TAZ. Consequently, YAP/TAZ is sequestered in the cytosol for proteasomal degradation.

YAP plays a significant role in mediating resistance to ferroptosis by downregulating GPX4 expression. Silencing YAP enhances ferroptosis, while its overexpression induces ferritinophagy by lowering mtROS and Fe^2+^ levels. Additionally, YAP1 disrupts the interaction between NCOA4 and FTH1, preventing ferritin degradation and Fe^2+^ release [60]. A transcriptome analysis identified the E3 ubiquitin ligase S-phase kinase-associated protein 2 (SKP2), a downstream target of YAP, as a regulator of ferroptosis. Reduced YAP levels decrease SKP2 expression, and both genetic and chemical SKP2 inhibition provide significant protection against ferroptosis. Suppression of YAP or SKP2 prevents lipid peroxidation during erastin-induced ferroptosis [61].

Moreover, ferroptosis can be regulated non-cell-autonomously through cadherin-mediated interactions. In epithelial cells, E-cadherin interactions inhibit ferroptosis by activating the NF2 (merlin) and Hippo signaling pathways. Disruption of this signaling allows YAP to enhance ferroptosis by increasing key modulators, such as ACSL4 and TfR2 [62].

### 2.8. MAPK Pathway

MAPK signaling pathway triggers downstream effector kinases or modulates transcription factor activity to elicit inflammatory and immune responses [63,64,65,66]. Intracellular iron overload and excessive lipid peroxidation, characteristic of ferroptosis, significantly affect MAPK activity. Importantly, these MAPKs interact to regulate cellular homeostasis, and their activation is linked to inflammation associated with ferroptosis. Inhibiting the MAPK signaling pathway could thus be a potential strategy for treating ferroptosis-related diseases, although more precise inhibitors need to be identified [67].

For instance, in a neonatal rat hypoxia-ischemia model, the Toll-like receptor 4 (TLR4)-p38 MAPK pathway promotes the production of proinflammatory cytokines IL-1β, IL-6, and IL-18 while decreasing the SLC7A11 and GPX4 expression, leading to neuroinflammation and ferroptosis [68]. Similarly, oxygen-glucose deprivation (OGD) activates this pathway, increasing malondialdehyde (MDA) levels and triggering ferroptosis in neuronal cells. Notably, the p38 inhibitor SB203580 can reduce OGD-induced ferroptosis by upregulating SLC7A11 and GPX4 [68].

Both p38 MAPK and ERK are involved in mediating inflammatory responses and ferroptosis. For instance, exposure to cadmium telluride quantum dots (CdTe QDs) promotes ferritinophagy via the Nrf2-ERK pathway, releasing iron from the LIP and triggering ferroptosis in macrophages [69]. Excessive iron accumulation can increase p38 and c-FOS phosphorylation, worsening hepatocellular injury.

Natural compounds like taxifolin mitigate oxidative stress and enhance hepatocellular survival by inhibiting MAPK activation [70].

Some agents, such as alpha-lipoic acid and resveratrol, act through the MAPK signaling pathway to exert anti-oxidant effects [71,72]. Conversely, mutations in the oncogene KRAS activate the MAPK pathway and promote resistance to ferroptosis in lung cancer cells [73], indicating that the MAPK pathway can significantly influence ferroptosis sensitivity in cancer contexts.

### 2.9. NF-κB

Nuclear factor-κB (NF-κB) is a family of transcription factors with five proteins: Rel (cRel), p65 (RelA), RelB, p105, and p100. p65, cRel, and RelB have a domain for DNA binding and dimerization, while p50 and p52 lack a transactivation domain. NF-κB forms dimers that bind to target genes, regulating their transcription [74]. NF-κB activation promotes the expression of cytokines and ROS, influencing apoptosis and chemoresistance in various cancer cells [74,75,76]. NF-κB can either promote or suppress ferroptosis, depending on tumor type, by regulating genes related to iron accumulation and lipid peroxidation [77]. Additionally, NF-κB/p65 counteracts the Nrf2-ARE pathway, influencing ferroptosis susceptibility. In HepG2 hepatoma cells, p65 depletion enhances Nrf2 activation of heme oxygenase-1 (HO1)-ARE, GPX2-ARE, and NAD(P)H dehydrogenase, quinone 1 (NQO1)-ARE-Luc reporters [78]. In glioblastoma cells, NF-κB activated by RSL3 increases lipid ROS and decreases GPX4, activating transcription factor 4 (ATF4) and SLC7A11; NF-κB combined with GPX4 depletion induces ferroptosis [79]. NF-κB p65 also prevents aspirin-induced ferroptosis in cancer cells by directly activating SCL7A11 [80].

## 3. Autophagy in Ferroptosis

Autophagy is a self-degradative process conserved throughout evolution that helps to maintain cellular homeostasis. It is mediated by the autophagosomes, which are double-layer membrane structures that mature by fusing with lysosomes for degradation [81,82]. Typically, autophagy acts as a defense mechanism, preventing the toxic buildup of damaged proteins and organelles that could lead to cancer development. However, in some cases, autophagy can aid cancer cells by supplying them with nutrients and energy needed for survival and cell growth [83]. The complex, context-specific interaction between autophagy and ferroptosis in cancer cells is still being explored. Autophagy can promote ferroptosis in cancer cells by breaking down iron-storage proteins like ferritin, releasing iron that triggers lipid peroxidation and oxidative stress. One study found that inducing autophagy in cancer cells increased the sensitivity of these cells to ferroptosis-inducing agents, such as erastin and RSL3 [84]. Consistently, loss of the tumor suppressor gene LKB1, which activates the AMP-activated protein kinase (AMPK) and inhibits mTOR, thereby activating autophagy, also enhances sensitivity to ferroptosis in lung cancer cells [50]. On the other hand, autophagy can also inhibit ferroptosis by removing damaged or oxidized lipids that would otherwise trigger the ferroptosis process. A previous study showed that blocking autophagy in liver cancer cells led to the accumulation of oxidized lipids and increased their resistance to ferroptosis [85]. Therefore, the influence of autophagy on ferroptosis in cancer cells depends on the specific context and conditions of the tumor microenvironment. However, the exact mechanisms by which autophagy regulates ferroptosis are still unclear and need further investigation. Gaining a deeper understanding of the relationship between autophagy and ferroptosis could pave the way for new therapeutic approaches for various diseases.

## 4. Epigenetic Control of Ferroptosis

**Figure 3:**
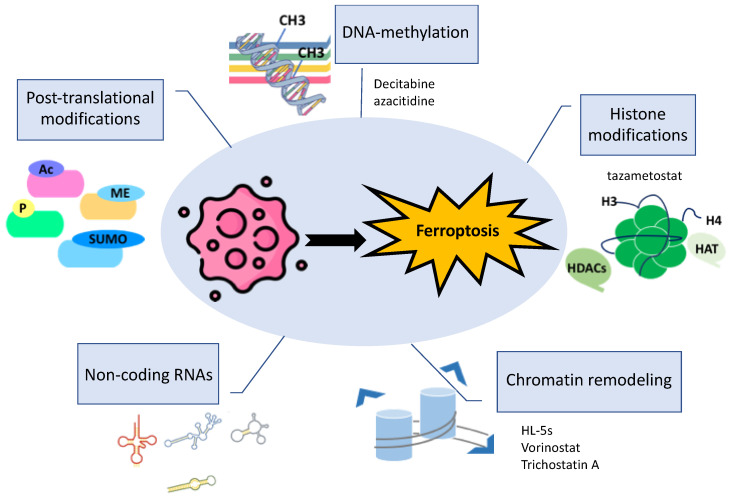
Schematic overview of the epigenetic mechanisms involved in the induction of ferroptosis in cancer cells. Experimental drugs targeting specific epigenetic modifications are also reported. CH3, methyl group; Ac, acetylation; Me, methylation; SUMO, sumoylation; P, phosphorylation; H3, histone H3; H4, histone H4, HAT, histone acetyltransferase; HDACs, histone deacetylases.

According to Deans and colleagues, the most suitable definition for epigenetics is “the study of phenomena and mechanisms that cause chromosome-bound, heritable changes to gene expression that are not dependent on changes to DNA sequence” [86]. Epigenetics, including histone modifications, DNA methylation, chromatin remodeling, and non-coding RNAs (Figure 3), are required mechanisms for a cell’s adaptability to various signals, conditions, and stressors [87]. Despite numerous studies, several epigenetic mechanisms and patterns remain unknown. Recent evidence has highlighted the crosslink between products of intermediary metabolism and chromatin proteins. These changes could be dynamic or stable and might even be inherited transgenerationally [88]. Therefore, these recent observations can correlate with complex phenomena such as cell death. Growing attention is being directed toward the epigenetic regulation of ferroptosis since it is closely related to metabolic dysregulation. Understanding the epigenetic regulatory mechanisms of ferroptosis in cancer and other diseases will pave the way for developing combination therapies that include epigenetic drugs and ferroptosis inducers. Such treatments could potentially help overcome cancer resistance to chemotherapy and prevent other diseases [89].

### 4.1. DNA Methylation

DNA methylation is an epigenetic modification that controls gene expression by adding a methyl group to the fifth carbon position of the cytosine base in mammals, resulting in the formation of 5-methyl-cytosine. This modification occurs predominantly at CpG sites, with approximately 60–90% of CpGs in the genome being methylated [90]. DNA methylation typically involves S-Adenosyl-methionine (SAM) as the methyl group donor and is primarily catalyzed by DNA methyltransferases (DNMTs) [91,92]. Research has shown that DNA methylation marks play a role in various biological processes and diseases, including the regulation of ferroptosis by influencing the expression of ferroptosis-related genes [93]. Generally, hypermethylation in a gene’s promoter or transcriptional start site of a gene, or hypomethylation within the coding region, tends to suppress that gene’s expression. However, the overall distribution of DNA methylation across the genome remains to be fully characterized [94]. Liu et al. analyzed data from the Cancer Genome Atlas data and found that DNA methylation may contribute to the varying expression levels of many ferroptosis regulator genes in tumors. They developed the ferroptosis potential index (FPI) and discovered that most cancer cells exhibit a high FPI compared to normal tissues, which predicted poor outcomes for several types of tumors [95]. Similarly, numerous DNA methylation signatures related to ferroptosis have been identified to forecast the prognosis in various cancers, including glioblastoma, head and neck squamous cell carcinoma, cutaneous melanoma, and lung squamous cell carcinoma [96,97,98,99]. Several iron-related genes, such as scavenger receptor class A member 5 (SCARA5), erythroferrone (ERFE), lipocalin 2, TfR2, SLC11A1, and cytochrome B reductase 1 (CYBRD1), were found to be dysregulated in different cancers, likely due to abnormal DNA methylation [99]. Moreover, iron overload can induce DNA demethylation, leading to the increased expression of Nrf2 target genes like NQO1 and GPX2, which subsequently promote cellular lipid peroxidation and ferroptosis [100]. PE-linked AA and AdA are substrates for lipid peroxidation. Fatty Acid Desaturase 1 (FADS1) and elongation of very long-chain fatty acid protein 5 (ELOVL5) are crucial for maintaining intracellular levels of AA and AdA. In intestinal-type gastric cancer cells, which exhibit resistance to ferroptosis, the expression of FADS1 and ELOVL5 is often downregulated due to heightened DNA methylation in their promoter and enhancer regions [101]. Additionally, GPX4, which is overexpressed in cancer tissues compared to normal tissues, is linked to decreased DNA methylation and an increase in H3K4me3 and H3K27ac marks at its promoter region, indicating that epigenetic regulation significantly contributes to the aberrant overexpression of GPX4 [102]. In upper gastrointestinal adenocarcinoma (UGC) tissue samples, increased methylation of CpG sites upstream of miR-4715-3p, which binds to and downregulates the 3′ UTR of Aurora kinase A (AURKA), was observed. Inhibition of AURKA by genetic knockdown or via reconstitution of miR-4715-3p led to a marked reduction of GPX4. Therefore, silencing of miR-4715-3p by DNA methylation promotes high expression levels of AURKA in UGCs, indicating that epigenetic regulation significantly contributes to the aberrant overexpression of GPX4 and protection against ferroptosis [103].

The Mucin 1 C-terminal subunit/CD44 variant (MUC1-C/CD44 var) complex directly interacts with and regulates xC, promoting its stability and controlling GSH levels. Additionally, MUC1 expression is finely regulated by histone and DNA methylations on its promoter [104]. In T- and B-acute lymphoblastic leukemia cell lines and patient samples, hypermethylation of the ferroptosis suppressor protein 1 (Fsp1) gene promoter prevents Nrf2 from binding and suppresses the expression of FSP1, a key enzyme involved in preventing lipid peroxidation, thereby promoting ferroptosis [105,106].

### 4.2. Histone Modifications

Histone modifications refer to the chemical changes made to the histone proteins around which DNA is wrapped, influencing gene expression by altering chromatin structure and accessibility.

Among the most common histone modifications, histone acetylation neutralizes histone’s positive charges and impairs their ability to bind DNA, leading to nucleosome depolymerization and gene transcriptional activation. Histone acetylation is regulated by the histone acetyltransferases (HATs), histone deacetylases (HDACs), and members of the bromodomain-containing protein (BRD) family. Whereas HATs add acetyl groups to histones, leading to a more open chromatin structure and increased gene expression, and HDACs remove these acetyl groups, resulting in chromatin condensation and reduced gene expression [107], the BRD family members recognize and bind to acetylation marks on histones, facilitating the modulation of chromatin structure and gene expression. In this context, ketamine, an inhibitor of lysine acetyltransferase 5 (KAT5), reduces H3K27ac levels at GPX4 promoter regions to promote ferroptosis in breast cancer [108]. In liver cancer, GSH production is regulated by hepatocyte nuclear factor 4 alpha (HNF4A) and the HIC ZBTB transcriptional repressor 1 (HIC1), which competitively binds to KAT2B. HNF4A has been identified as a ferroptosis inhibitor, whereas HIC1 acts as a ferroptosis inducer [109]. Further studies are needed to explore the cell-specific expression mechanisms of HDACs and to elucidate how histone acetylation modifications precisely influence ferroptosis.

Methylation is another important histone modification that mainly occurs at the N-terminal lysine (K) or arginine (R) residues in histones H3 and H4. Among these modifications, H3K4me1/2/3, H3K36me1/2/3, and H3K79me1/2/3 are typically associated with transcriptional activation, whereas H3K9me3, H3K27me3, and H4K20me2/3 generally function to repress transcription [110,111,112,113]. Overall, GPX4 overexpression in tumor cells is partly due to the accumulation of H3K4me3 at its gene promoter [102]. In gastric cancer, the enzyme methionine adenosyltransferase 2A (MAT2A) enhances the production of the methylation donor SAM, which increases the levels of H3K4me3 at the promoter of ACSL3, subsequently inhibiting ferroptosis [114]. As previously noted, in breast cancer, the transmembrane oncoprotein MUC1-C binds to a CD44 variant, stabilizing the SLC7A11 molecule. This interaction is regulated by H3K9me2/3 modifications at the MUC1-C promoter, which downregulates MUC1-C gene transcription and, in turn, affects GPX4′s capacity to induce ferroptosis [104].

On the other hand, histone demethylases, which remove methyl groups from modified histones, play a critical role in ferroptosis. For instance, lysine demethylase 4A (KDM4A) reduces the levels of H3K9me3 at the SLC7A11 promoter, leading to increased expression of SLC7A11 in osteosarcoma [115]. Additionally, in clear cell renal cell carcinoma, inhibiting the histone methyltransferase SUV39H1, responsible for adding H3K9me3 marks, protects cells from ferroptosis. Specifically, SUV39H1 targets the promoter region and suppresses the expression of dipeptidlypeptidase-4 (DPP-4), which interacts with NADPH oxidase 1 (NOX1) to promote lipid peroxidation [116].

### 4.3. Chromatin Remodeling and Interplay with Transcription Factors

While transcription factors themselves are not epigenetic regulators, they work closely with the epigenetic machinery to regulate gene expression. For example, transcription factors can recruit epigenetic modifiers to specific genes, leading to increased gene expression.

High mobility group AT-hook 1 (HMGA1) is classified as an “architectural transcription factor” that plays a crucial role in remodeling chromatin structure and regulating transcription factor-DNA interactions. HMGA1 has been implicated in cellular transformation and is frequently upregulated in both spontaneous and carcinogen- or viral oncogene-induced tumors [117,118]. HMGA1 also regulates ferroptosis-related genes, and its specific role in controlling ferroptosis in esophageal cancer has been recently highlighted [119]. Yang et al. demonstrated that HMGA1 facilitates the recruitment and binding of the transcriptional factor ATF4 to the SLC7A11 promoter, thereby enhancing its transcription. Consequently, depletion of HMGA1 promotes ferroptosis and restores the sensitivity of esophageal squamous cell carcinoma (ESCC) to chemotherapy both in vitro and in vivo. These findings underscore the critical role of HMGA1 in repressing ferroptosis and promoting cisplatin resistance in ESCC. Therefore, targeting HMGA1 represents a potential strategy to overcome ESCC chemoresistance by inducing ferroptosis [119]. HMGA2, another oncogenic member of the HMGA protein family, also plays a key role in preventing ferroptosis in pancreatic cancer cells by enhancing both transcription and stabilization of GPX4 [120]. However, the regulation of GPX4 by HMGA2 appears more complex in prostate cancer, where the truncated HMGA2 downregulates, rather than upregulates, GPX4 [121]. The AT-rich interactive domain-containing protein 1A (ARID1A) is a key component of the switch/sucrose nonfermenting (SWI/SNF) chromatin-remodeling complex, which promotes Nrf2-mediated transcriptional activation of SLC7A11. In contrast, ARID1A-deficient cancer cells exhibit heightened vulnerability to the inhibition of the antioxidant GSH and targeting of glutamate-cysteine ligase catalytic subunits [122]. Moreover, lymphoid-specific helicase (LSH), belonging to the SNF2 helicase family of chromatin-remodeling proteins, promotes the transcription of ferroptosis repressors, such as FADS2 and stearoyl-CoA desaturase 1 (SCD1). LSH is negatively regulated by HIF1A, indicating that it can quickly respond to hypoxic environments and dynamically regulate target genes [123,124]. Wang and colleagues have recently reported that LSH is able to enhance the expression of SLC7A11 by binding to its promoter region, playing a critical role in tumor development by inhibiting ferroptosis [125]. This finding suggests a promising strategy for developing anti-cancer therapeutics by targeting metabolic properties. However, further exploration is needed into other chromatin remodeling regulators, such as Imitation Switch (ISWI) and chromodomain helicase DNA (CHD), and their role in ferroptosis [126].

### 4.4. Non-Coding RNAs

Non-coding RNAs (ncRNAs) make up approximately 98% of the transcriptome [127]. Based on their length and structure, ncRNAs are categorized into several types, including microRNAs (miRNAs), PIWI-interacting RNAs (piRNAs), small nuclear RNAs (snRNAs), small nucleolar RNAs (snoRNAs), long ncRNAs (lncRNAs), circular RNAs (circRNAs), transfer RNAs (tRNAs), and ribosomal RNAs (rRNAs) [128,129]. Although they play a significant role in regulating ferroptosis by influencing several key processes, including the regulation of mitochondrial-related proteins, iron metabolism, GSH metabolism, and lipid peroxidation [130], the role of ferroptosis-related ncRNAs in cancer remains largely unclear [131].

CircRNAs are covalently closed, single-stranded RNA molecules derived from exons through alternative mRNA splicing, also playing a role in regulating ferroptosis [131]. In glioma, the circular RNA Tau tubulin kinase 2 (Circ-TTBK2) promotes cell proliferation and invasion while inhibiting ferroptosis by sponging miR-761 and activating Integrin Subunit Beta 8 (ITGB8). Accordingly, the knockdown of circ-TTBK2 promotes erastin-induced ferroptosis [132]. In cervical cancer cells, high-throughput microarray-based circRNA profiling identified 526 dysregulated circRNAs, with bioinformatic analyses indicating their primary involvement in GSH metabolism. However, further research is needed to explore how circRNAs modulate ferroptosis, as the associated miRNAs and downstream factors were not examined in this study [133].

LncRNAs are broadly defined as cell endogenous non-coding RNA molecules that are longer than 200 nucleotides. They serve as signals, decoys, guides, and scaffolds for the cellular components to perform multiple functions [134]. Regulation of GSH by lncRNAs in cancer mainly depends on the enzymes GSH S-transferase (GST) and Glutamate-cysteine ligase (GCL). In leukemia cells, ferritin heavy chain (FHC) silencing leads to ROS production and alters downstream genes via the increase in lncRNA H19 and miR-657 expression. This means that lncRNAs are associated with iron metabolism in cancer cells [135]. Moreover, lncRNAs can regulate the transcription factor NRF2 either directly, by controlling its expression, or indirectly, by modulating Kelch-like ECh-Associated Protein 1 (KEAP1). Additionally, in bladder cancer, the lncRNA urothelial cancer associated 1 (Uca1) reduces ROS levels by targeting miR-16, which in turn decreases GSH synthetase levels. This regulation contributes to glutamine metabolism and to the maintenance of the redox state [136]. In malignant melanoma, knockdown of the lncRNA growth arrest-specific 5 (GAS5) led to increased intracellular ROS accumulation by elevating superoxide anion levels and promoting NADPH oxidase 4 (NOX4)-mediated GSH oxidation. Furthermore, RNA co-immunoprecipitation showed that GAS5 induces these changes by interacting with glucose-6-phosphate dehydrogenase (G6PD) [137]. In HCC, the inhibition of H19 triggers oxidative stress and reduces the chemoresistance of CD133+ cancer stem cells by blocking the MAPK/ERK signaling pathway [138]. In lung cancer cells, overexpression of the lncRNA maternally expressed gene 3 (MEG3) induces cell death and increases sensitivity to paclitaxel in a ROS-dependent manner. Conversely, MEG3 knockdown reduces the intracellular oxidative stress induced by paclitaxel [139].

miRNAs are short RNA molecules from 19 to 25 nucleotides in size that regulate the posttranscriptional silencing of target genes. A single miRNA can target hundreds of different mRNAs and influence the expression of many genes often involved in a functional interacting pathway [140]. Recent literature has highlighted the connection between miRNAs and ferroptosis. Several miRNAs have been characterized as having either anti- or pro-ferroptosis properties, depending on their specific targets. Given that the antioxidant capacity, peroxide tone, and iron availability are fundamental modulators of ferroptosis, miRNAs act as fine-tuning regulators of these processes.

According to Tomita et al., miR-7-5p inhibits ferroptosis by targeting and downregulating mitoferrin, thereby reducing mitochondrial Fe^2+^ levels and decreasing the occurrence of ferroptosis [141]. Furthermore, in melanoma cells, miR-9 regulates ferroptosis by directly inhibiting glutamic oxaloacetic transaminase 1 (GOT1), which plays a critical role in amino acid metabolism, particularly in the metabolism of glutamine, a key factor in ferroptosis [142]. miR-6852, regulated by lncRNA Linc00336, inhibits the growth of lung cancer cells by promoting ferroptosis. This is achieved through its regulation of cystathionine-β-synthase (CBS), a key enzyme involved in cysteine production and a marker of ferroptosis [143]. Finally, the connection between miRNAs and key ferroptosis regulators such as GSH, iron, and NRF2 in cancer cells is also well documented [131,144,145,146,147,148].

### 4.5. Post-Translational Modifications

Post-translational modifications (PTMs), including phosphorylation, acetylation, ubiquitination, methylation, and SUMOylation, which can be reversible, play a crucial regulatory role in ferroptosis [149,150]. PTMs are chemical modifications of specific amino acids that affect the conformation, activity, interaction, stability, and spatial distribution of most eukaryotic proteins. They act as a switch, allowing cells or organisms to rapidly and precisely respond to external stimuli, including stress [151]. Maintaining proper protein modification homeostasis is crucial for human health. Abnormal PTMs can lead to changes in protein properties and loss of protein function. These changes are closely associated with the development and progression of many diseases [152]. Because most PTMs are reversible, normal cells use PTMs as a switch to decide the cell’s static and proliferative state. In cancer cells, the activation of oncogenes and/or inactivation of tumor suppressor genes provide continuous proliferation signals by modulating the diverse PTM states of effector proteins involved in cell survival, cell cycle, and proliferation. This dysregulation leads to abnormal cancer cell proliferation.

Phosphorylation is the most common PTM, which typically occurs on serine, tyrosine, and threonine residues of the targeted protein [149]. In mouse embryonic fibroblasts, Lee et al. showed that glucose deprivation inhibits ferroptosis induced by erastin. AMPK, which serves as a key sensor of cellular energy levels, becomes phosphorylated and activated in response to glucose starvation. Mechanistically, AMPK phosphorylates and inactivates acetyl-CoA carboxylase, leading to reduced biosynthesis of PUFAs and other fatty acids, thereby inhibiting ferroptosis. This pathway mediates the inhibitory effect of glucose starvation on ferroptosis. Moreover, cancer cells with high basal AMPK activation are resistant to ferroptosis, while AMPK inactivation sensitizes them to ferroptosis. These findings suggest that AMPK acts as a negative regulator of ferroptosis [50]. On the contrary, AMPK-mediated phosphorylation of BECN1 at Ser90/93/96 enhances ferroptosis by binding to SLC7A11 and directly inhibiting the system Xc^−^. Silencing BECN1 suppresses ferroptosis induced by the system Xc^−^ inhibitors such as erastin, sulfasalazine, and sorafenib. Therefore, the phosphorylation and activation of the BECN1 pathway promote ferroptosis in cancer cells [153]. In HCC, Akt, activated by insulin growth factor receptor 1, phosphorylates creatine kinase B at T133, which decreases its metabolic activity and increases its binding to and phosphorylation of GPX4 at S104. This interaction prevents the heat shock protein 70 from binding to GPX4, thereby inhibiting the degradation of GPX4 via chaperone-mediated autophagy, which in turn inhibits ferroptosis and supports tumor growth in mice [154].

Metazoan spot homolog 1 (MESH1) is a cytosolic NADPH phosphatase that promotes ferroptosis by directly degrading the essential metabolite NADPH. MESH1, as an NADPH phosphatase, plays a crucial role in ferroptosis by depleting NADPH and impairing GSH regeneration, key processes in the execution of ferroptosis [155]. Activation of the protein kinase C βII (PKCβII) is essential for the execution of ferroptosis, with lipid peroxidation being necessary for PKCβII activation. PKCβII directly interacts with and phosphorylates ACSL4 at Threonine 328, leading to ACSL4 activation and triggering the biosynthesis of PUFA-containing lipids. This promotes the generation of lipid peroxidation products. In turn, the lipid peroxidation–PKCβII–ACSL4 positive-feedback loop drives ferroptosis by amplifying lipid peroxidation to lethal levels [156].

Acetylation typically occurs on lysine residues of target proteins, with acetyltransferases transferring acetyl groups from acetyl coenzyme A to these residues, effectively neutralizing their charges. This modification is reversible and can be undone by deacetylases. Protein acetylation plays a vital role in various cellular processes related to physiology and diseases, including transcriptional activity, protein stability, enzyme function, protein–protein interactions, subcellular localization, and protein–DNA interactions [157]. p53 is a crucial regulator of ferroptosis, and its function is significantly influenced by acetylation [131,158,159]. Specifically, p53 reduces SLC7A11 expression to lower cystine uptake, making cells more sensitive to ferroptosis. Moreover, the acetylation-defective mutant p53^3KR^ (K117R, K161R, and K162R) maintains its ability to suppress SLC7A11 expression but is unable to induce cell cycle arrest, senescence, or apoptosis [160]. Further research has shown that the p53^4KR^ (K98R + 3KR) mutant completely loses its ability to regulate SLC7A11, whereas the p53^K98R^ mutant alone has only a modest effect on p53-mediated transactivation [161]. Ferroptosis cells release High-mobility group box 1 (HMGB1), a damage-associated molecular pattern (DAMP) molecule, in an autophagy-dependent manner. Additionally, inhibition of HDACs through autophagy facilitates the acetylation and subsequent release of HMGB1 [162].

Protein methylation is another common type of PTM, comparable in prevalence to phosphorylation and ubiquitination. Protein methylation takes place on lysine or arginine residues in both histone and nonhistone proteins [163,164]. While histone methylation is the most prevalent form of protein methylation, numerous non-histone proteins, including p53 and Akt, have also been found to undergo methylation. The methylation of non-histone proteins not only influences their activity and stability but also interacts with other PTMs to regulate their function, including in cancer contexts [165,166,167]. Recently, the significance of protein methylation in ferroptosis has gained greater attention and emphasis [168]. Lysine demethylase 3B (KDM3B) enhances the expression of SLC7A11 by interacting with ATF4, thereby inhibiting ferroptosis induced by erastin [169]. Knockdown of BRD4 and treatment with (+)-JQ1, a BRD4 inhibitor, both induce ferroptosis in breast cancer cell lines through ferritinophagy. (+)-JQ1 inhibits the expression of euchromatic histone lysine methyltransferase 2 (EHMT2, also known as G9a) while promoting SIRT1 expression to suppress BRD4. These effects downregulate the expression of GPX4, SLC7A11, and SLC3A2, thereby regulating ferritinophagy [170]. Recent literature indicates that H3K9 methylation is a key epigenetic mechanism regulating ferroptosis. Targeting regulators of H3K9 methylation can significantly inhibit ferroptosis and hold promise as a potential treatment for degenerative diseases characterized by cell loss. Conversely, enhancing H3K9 methylation may offer therapeutic benefits for conditions with excessive cell proliferation, such as tumors, by promoting ferroptosis [94].

Ubiquitination, also referred to as ubiquitylation, is a type of PTM where ubiquitin is attached to a target protein. Ubiquitin is a 76-amino acid protein that can exist as a free molecule or be conjugated to other proteins in a single unit (monoubiquitination) or as a chain of multiple ubiquitins (polyubiquitination). Ubiquitination has a different range of effects on protein function, influencing processes such as protein degradation, subcellular localization, and kinase activation. It is also significantly implicated in the pathobiology of various human diseases [171]. For instance, cancer cells treated with palladium pyrithione complex (PdPT), which acts as a pan-deubiquitinase (pan-DUB) inhibitor, experience apoptosis and ferroptosis characterized by caspase activation and degradation of the GPX4 protein [172]. However, the mechanisms underlying GPX4 ubiquitination and the specific ubiquitination sites need further investigation. In cancer cells, BRCA1-associated protein 1 (BAP1) removes monoubiquitin from ubiquitinated H2A at lysine 119 (H2Aub) on the SLC7A11 promoter, thereby suppressing its expression in cells treated with erastin and activating ATF4 [173,174]. The reduction of SLC7A11 levels inhibits cystine uptake, leading to increased lipid peroxidation and ferroptosis. Wang et al. observed lower levels of monoubiquitination of histone H2B on lysine 120 (H2Bub) in cells induced by erastin compared to control cells. Further investigation revealed that the tumor suppressor p53 facilitates the nuclear translocation of the deubiquitinase ubiquitin-specific peptidase 7 (USP7), which negatively regulates the H2Bub levels in a transcriptional-independent manner. This action decreases SLC7A11 expression during erastin treatment, thereby promoting ferroptosis [159]. Additionally, the androgen receptor (AR), a steroid hormone receptor and a well-recognized biomarker for predicting prognosis in prostate cancer, is targeted by ALZ003, a curcumin analog. ALZ003 enhances the survival of transplanted mice by inducing ferroptosis in glioblastoma cells. This effect is mediated by the F-box and the leucine-rich repeat protein 2- (FBXL2-)-dependent ubiquitination and subsequent degradation of AR [175].

## 5. Epigenetic and Therapeutic Strategies for Targeting Ferroptosis in Cancer

Ferroptosis offers a method for selectively targeting tumor cells, improving the effectiveness of drug-induced cell death while minimizing harmful effects on normal cells [176]. As discussed earlier, ferroptosis plays a crucial role in tumor biology. On one hand, the induction of ferroptosis is essential for the function of tumor suppressors. On the other hand, cancer cells have developed various strategies to evade ferroptosis, thereby enhancing their survival. Notably, mesenchymal and dedifferentiated cancer cells, which often resist apoptosis and standard treatments, demonstrate a significant vulnerability to ferroptosis. As a result, ferroptosis is becoming a promising target for cancer therapy, especially in challenging cases of resistant tumors [176]. Epigenetic drugs can be useful for targeting cancer because they can reverse abnormal gene expression patterns without altering the DNA sequence itself. By targeting these reversible modifications, epigenetic drugs offer a way to “reprogram” cancer cells to a more normal state or make them more susceptible to existing therapies. Drugs that modulate ferroptosis in cancer from an epigenetic perspective, although research in this area is still emerging, are a valuable possibility. The molecules involved in the epigenetic regulation of ferroptosis include HDAC inhibitors, enhancer of zeste homolog 2 (EZH2) inhibitors, non-coding RNA modulators, and DNMT inhibitors.

HDAC inhibitors, such as HL-5s, vorinostat, and trichostatin A, can affect the sensitivity of cancer cells to ferroptosis by epigenetically regulating the expression of genes involved in iron metabolism and oxidative stress. Modulation of histone deacetylation can alter the activity of genes that either protect against or sensitize cancer cells to ferroptosis. A novel Benzimidazole Derivative, HL-5s, has robust inhibitory activity against class I HDACs, particularly HDAC1. Notably, it induces ferroptosis by augmenting lipid peroxidation production. Mechanistically, HL-5s increased the YB-1 acetylation and inhibited the NRF2/HO-1 signaling pathway [177]. Suberoylanilide hydroxamic acid, also known as vorinostat, promotes ferroptosis via downregulation of the glutamate-cystine antiporter xCT. This has been shown therapeutic effects in *EGFR*-activating mutant lung cancer cells that display intrinsic or acquired resistance to EGFR-TKI [178]. Trichostatin A promotes the cytotoxicity of the genotoxic anticancer drug cisplatin. Zhou et al. recently showed that in the presence of TSA, cisplatin downregulated the mitochondrial transcription factor A (TFAM) and SLC3A2 to enhance cisplatin-induced ferroptosis, also contributing to the promotion of cisplatin cytotoxicity [179].

Similarly, EZH2 inhibitors, such as tazemetostat, can influence the expression of ferroptosis-related genes, modulating iron metabolism and oxidative balance in cancer cells [180]. Mechanistically, EZH2 amplifies the modification of H3K27 me3, thereby downregulating TfR2 expression and suppressing ferroptosis. The combination of tazemetostat with sorafenib demonstrated significant synergistic ferroptosis-promoting effects in HCC cells resistant to this drug [180]. On the other hand, EZH2 inhibitors impair the occurrence of ferroptosis by upregulating the heat shock protein family A member 5 (HSPA5) and stabilizing GPX4 in diffuse large B-cell lymphoma (DLBCL). However, co-treatment with ferroptosis inducer erastin effectively overrode the resistance of DLBCL to EZH2 inhibitors in vitro and in vivo [181].

As reported above, several miRNAs and lncRNAs regulate ferroptosis-related processes through epigenetic mechanisms. Prognostic models based on the expression patterns of ferroptosis lncRNAs have been reported in pancreatic ductal carcinoma, gastric cancer, and melanoma [182,183,184]. Modulation of these non-coding RNAs could influence ferroptosis activity, inducing cell death in apoptosis-resistant cancers [185].

Drugs that inhibit DNMTs, such as decitabine or azacitidine, can induce ferroptosis in certain cancer types by affecting the epigenetic regulation of key genes involved in iron metabolism and antioxidation [186]. By demethylating and downregulating *SLC7A11*, decitabine may impair cystine uptake, reducing GSH levels and increasing susceptibility to ferroptosis [187]. In head and neck cancer (HNC) cells with low expression of E-cadherin (CDH1), the treatment of 5-azacitidine diminishes the hypermethylation of CDH1, resulting in increased E-cadherin expression and decreased ferroptosis susceptibility, thereby suggesting that epigenetic reprogramming of EMT contributes to promoting ferroptosis in HNC [188].

Integrating epigenetic drugs with therapies that induce ferroptosis represents a promising therapeutic strategy to sensitize resistant cancer cells and enhance the efficacy of cancer treatments.

Different molecules, as summarized in Table 1, have been shown to induce ferroptosis in cancer cells. Sorafenib, the first tyrosine kinase inhibitor authorized for treating unresectable HCC, advanced renal cell carcinoma, and differentiated thyroid cancer, has been found to induce ferroptosis by blocking the system Xc^−^ and elevating intracellular iron levels. Sorafenib-induced ferroptosis not only directly eliminates cancer cells but also offers further therapeutic advantages, highlighting its multifaceted role as a treatment option. As a ferroptosis inducer, Sorafenib can diminish the stemness of gastric cancer cells [189]. Additionally, it can initiate ferroptosis to enhance the sensitivity of melanoma cells to Vemurafenib, an FDA-approved B-Raf kinase inhibitor for advanced melanoma [190]. Furthermore, Sorafenib may represent a novel treatment strategy for patients with cisplatin-resistant non-small cell lung cancer (NSCLC) by activating ferroptosis [191].

Neratinib, another tyrosine kinase inhibitor that targets HER2 (human epidermal growth factor receptor 2) and, to a lesser extent, EGFR (epidermal growth factor receptor), promotes ferroptosis and suppresses brain metastasis in HER2-positive breast cancer when used as neoadjuvant therapy, primarily by increasing intracellular iron levels [192]. Moreover, Ma et al. demonstrated that neratinib induces ferroptosis in acute myeloid leukemia (AML) cells, as evidenced by increased ROS levels, elevated MDA content, enhanced cellular Fe^2+^ activity, and downregulation of GPX4 and FTH1 expression, along with upregulation of ACSL4 expression. Therefore, neratinib inhibits AML cell proliferation by activating autophagy-dependent ferroptosis [193].

Recent studies highlighted that cisplatin can also induce ferroptosis in HCT116 and A549 cancer cell lines by reducing GSH and inactivating GPX4, offering an alternative mechanism for inhibiting tumor growth [194]. Sulfasalazine (SAS), an anti-inflammatory medication commonly prescribed for rheumatoid arthritis, has been shown to induce ferroptosis by inhibiting the cystine/glutamate antiporter SLC7A11. Like other inhibitors of the system Xc^−^, SAS effectively triggers ferroptosis cell death in chemotherapy-resistant cells and enhances their responsiveness to chemotherapy [195,196,197].

Another class of ferroptosis-inducing drugs is statins, commonly used for reducing blood cholesterol levels [198]. Statins block the GSH/GPX4 and FSP1/CoQ_10_/NAD(P)H axes via the mevalonate pathway, thus inducing ferroptosis [199]. Among statins, lovastatin converts the immuno-cold phenotype into an inflammatory one and promotes ferroptosis in NSCLC by downregulating PD-L1 expression in lung cancer cells, thereby enhancing tumor responsiveness to immunotherapy [200].

Artemisinin is a derivative of the herb *Artemisia annua*. It has anti-tumor effects in several tumors via ferroptosis [201]. The increase in ferritinophagy and intracellular free iron levels are the mechanisms underpinning its activity, which finally leads to ferroptosis and tumor inhibition [202].

Haloperidol, an antagonist of dopamine receptor D2, commonly used for the treatment of psychiatric disorders [203], exhibits anticancer effects by inducing ferroptosis, but the precise mechanism remains unclear. It may involve a potential link with autophagy. In fact, haloperidol shows synergistic activity with temozolomide in inhibiting glioblastoma (GBM) growth by enhancing temozolomide-induced autophagy-mediated ferroptosis [204].

Zalcitabine, commonly used for the treatment of patients infected with the human immunodeficiency virus, acts by targeting mitochondrial DNA polymerase gamma [205]. Mechanistically, zalcitabine induces oxidative mitochondrial DNA damage and decreased mitochondrial function, as well as degradation of TFAM. These effects result in the activation of the DNA damage-sensing CGAS-STING1 pathway, inducing autophagy and subsequent lipid peroxidation-mediated ferroptosis [206].

β-Elemene (β-ELE) is a noncytotoxic broad-spectrum antitumor agent derived from *Curcuma wenyujin* that exhibits noncytotoxic properties and is commonly used in the treatment of NSCLC. This natural substance interacts with the transcription factor TFEB, which plays a crucial role in lysosome biogenesis, promoting the TFEB-mediated degradation of GPX4 in lysosomes. This process induces ferroptosis in NSCLC, leading to tumor suppression [207].

Withaferin A (WA), a bioactive compound extracted from *Withania somnifera* roots, effectively targets and inactivates GPX4, resulting in the elimination of high-risk neuroblastoma and a reduction in relapse rates by inducing ferroptosis [208]. Furthermore, WA has been shown to increase the expression of KEAP1, which reduces downstream NRF2 levels in both parental and Sorafenib-resistant HCC cells. This process attenuates metastatic potential and induces ferroptosis [209].

Buthionine sulfoxide amine (BSO) effectively inhibits the de novo synthesis of GSH, inactivating GPX4 and promoting lipid peroxidation accumulation by blocking its conversion to lipid alcohols. This dual mechanism leads to a high rate of ferroptosis in murine breast cancer cells [210].

Brequinar, an inhibitor of DHODH, works alongside mitochondrial GPX4 (independently of cytosolic GPX4) to inhibit ferroptosis in the mitochondrial inner membrane. It does this by reducing ubiquinone to ubiquinol, a radical-trapping antioxidant that has anti-ferroptosis activity. Brequinar is a potential agent for treating cancers with low expression of GPX4 (GPX4^low^) by triggering ferroptosis. In contrast, the combined administration of brequinar and sulfasalazine synergistically suppresses the growth of tumors with high GPX4 expression (GPX4^high^) [211].

Curcumenol, an effective compound found in Wenyujin, separated from the volatile oil, has been demonstrated to exert antitumor activity by triggering ferroptosis both in vitro and in vivo in lung cancer cells by activating the lncRNA H19/miR-19b-3p/FTH1 axis, essential for curcumenol-induced ferroptotic cell death [212].

**Table 1:** Main therapeutic drugs targeting ferroptosis.

Drug	Ferroptosis Effects	Cancer Type	Tumor Effects	References
Sorafenib	Inhibition of the System Xc^−^	Gastric cancer	Cancer stem cell reduction	[189]
		Melanoma	Overcoming chemotherapy resistance	[190]
		NSCLC	Overcoming chemotherapy resistance	[191]
Neratinib	Increase in intracellular iron levels	Breast cancer	Suppression of brain metastasis	[192]
	Inhibition of anti-oxidant defense	Acute myeloid leukemia	Induction of ferroptosis-dependent autophagy	[193]
Cisplatin	Increase in intracellular iron levels Generation of ROS GSH reduction Inhibition of GPX4 Up-regulation of ACSL4	Lung cancer Colon cancer	Inhibition of tumor growth Overcoming apoptosis resistance	[194]
Sulfasalazine	Inhibition of the System Xc^−^	Lymphoma	Inhibition of tumor growth	[195]
		Breast cancer		[196]
		Head and neck cancer		[197]
Lovastatin	GSH/GPX4 and FSP1/CoQ_10_/NAD(P)H axes inhibition	NSCLC	Enhanced immunotherapy sensitivity	[197]
Artemisinin	Increase in ferritinophagy and intracellular free iron levels	NSCLC Colon cancer Renal cancer Ovarian cancer CNS tumor Leukemia Melanoma Prostate carcinoma Breast cancer	Overcoming chemotherapy resistance	[202]
Haloperidol	Increase in ROS levels Increase in intracellular iron levels Increase in PUFA GSH reduction Inhibition of GPX4	GBM	Inhibition of tumor growth	[204]
Zalcitabine	Increase in mitochondrial damage Increased TFAM degradation	Pancreatic ductal adenocarcinoma	Inhibition of tumor growth Induction of ferroptosis-dependent autophagy	[206]
β-Elemene	Activation of TFEB-mediated lysosome degradation of GPX4	NSCLC	Tumor suppression	[207]
Withaferin	GPX4 inactivation	Neuroblastoma	Suppression of tumor relapse	[208]
	Upregulation of Keap1 and downregulation of Nrf2	HCC	Attenuation of metastatic potential; Overcoming sorafenib resistance	[209]
Buthionine sulfoxide amine (BSO)	GSH reduction GPX4 inactivation LPO accumulation	Breast cancer	Overcoming chemotherapy resistance	[210]
Brequinar	Reduction of anti-oxidant defense by inhibition of CoQ enzyme Increase in ROS levels	Clear cell carcinoma Renal cell carcinoma	Inhibition of tumor growth	[211]
Curcumenol	lncRNA H19/miR-19b-3p/FTH1	Lung cancer	Tumor suppression	[212]

## 6. Conclusions

Ferroptosis is a regulated cell death linked to metabolic disruption in iron homeostasis, fatty acid synthesis, and lipid oxidation. Ferroptosis has emerged as a fundamental cellular death mechanism in diseases and pathological conditions. In this review, we described the molecular mechanisms underpinning ferroptosis, the complex interplay between epigenetic mechanisms and ferroptosis, as well as the potential strategies to enhance therapeutic efficacy and overcome resistance in cancer therapy by harnessing the therapeutic potential of ferroptosis. Albeit this goal holds great expectations as an anticancer strategy, several additional challenges remain to be overcome in future research. Standardized animal models are needed to deepen knowledge about ferroptosis biology in cancer and ease the comparison of studies between research laboratories and clinicians. Several compounds endowed with the ability to induce ferroptosis show poor bioavailability and insufficient targeting. Moreover, since cell sensitivity depends on tumor origin and genotype, the multiple genetic information from the cancer genome needs to be integrated for predicting tumor response to specific ferroptosis drugs [176]. Hence, more detailed mechanistic insights are needed to overcome these challenges to improve strategies and combat the “rust” of therapy-resistant cancers as a stepping stone toward translation into the clinic.

Looking to the future, exploring combination therapies that integrate ferroptosis inducers with existing anti-cancer agents offers a promising path to amplify therapeutic outcomes. Another exciting perspective lies in the development of nanoparticle-based drug delivery systems, which could enhance the bioavailability and targeted delivery of ferroptosis-inducing compounds. These innovations could help address current limitations, paving the way toward effective clinical applications of ferroptosis-targeted treatments in oncology.

## Data Availability

Not applicable.
